# Advance Glycation End Products Inhibition by *Citrus paradisi* Peel Extract; Characterization, LCMS‐QTOF Analysis, and Biological Evaluation

**DOI:** 10.1002/fsn3.4602

**Published:** 2024-11-21

**Authors:** Muhammad Fakhar Ul Mehmood, Marvi, Hatem A. Abuelizz, Nighat Aziz, Raheela Bano, Asif Wazir, Imran Ahmad, Khizar Abbas, Saiqa Ishtiaq, Adnan Amin

**Affiliations:** ^1^ Department of Pharmacology, Faculty of Pharmacy University of Balochistan Quetta Pakistan; ^2^ NPRL, Department of Pharmacognosy, Faculty of Pharmacy Gomal University Dera Ismail Khan Khyber Pakhtunkhwa Pakistan; ^3^ Department of Pharmaceutical Chemistry, College of Pharmacy King Saud University Riyadh Saudi Arabia; ^4^ Department of Pharmacology Gomal Medical College Dera Ismail Khan Khyber Pakhtunkhwa Pakistan; ^5^ Department of Pathology Gomal Medical College Dera Ismail Khan Khyber Pakhtunkhwa Pakistan; ^6^ Department of Pharmacognosy Bahauddin Zakariya University Multan Pakistan; ^7^ Department of Pharmaceutical Chemistry Bahauddin Zakariya University Multan Pakistan; ^8^ Centre for Study of Human Health Emory University Atlanta Georgia USA

**Keywords:** AGEs, antioxidant, Citrus *Spp.*, medium polar, polymethoxylated flavonoids

## Abstract

Advance glycation end products (AGEs) are the main reason for diabetic complications. Persistent hyperglycemia and non‐enzymatic glycation increase the rate of AGEs formation. Natural functional food‐based approaches are mainly under investigation these days to discover new treatment options. We focused to investigate potential of medium polar fractions of *Citrus paradesi*. The peels fractions were analsyed for phytochemical profile, FTIR, HPLC‐DAD, and LCMS‐QTof, and biological investigation including antioxidant assays, α‐glucosidase inhibition, and Anti AGEs inhibition was performed. LCMS‐QTof confirmed the presence of flavonoids and polymethoxylated flavonoids including naringin, narirutin 4‐O‐glucoside, hesperidin, naringenin‐7‐O‐rutinoside hexamethoxyflavone, 3,5,6,7,8,3′,4′ heptamethoxyflavone were major compounds. A significant antioxidant activity was recorded in case of chloroform fraction compared to ethyl acetate fraction. Similarly a substancial AGEs inhibition in oxidative mode (IC_50_ 0.23 mg/mL) and non‐oxidative mode (IC_50_ 0.10 mg/mL) was observed in chloroform fraction, whereas ethyl acetate fraction was only active in oxidative mode (IC_50_ 0.69 mg/mL). A moderate α‐glucosidase inhibition (IC_50_ 1.23 mg/mL) was noticed in total extract, while significant activity was recorded in chloroform fraction (IC_50_ 0.78 mg/mL). It was concluded that medium polar fraction of *C. paradesi* possesses antidiabetic and anti‐AGEs potential that can be due to presence of flavonoids and polymethoxylated flavonoids.

## Graphical abstract

The citrus peel is a rich source of PMFs and they are provided by strong antioxidant potential. The poly methoxy groups are interesting and contribute mainly towards Biological activities. Herein the citrus peel extracts presented significant AGEs inhibition that can employed for management of Diabetic complications.

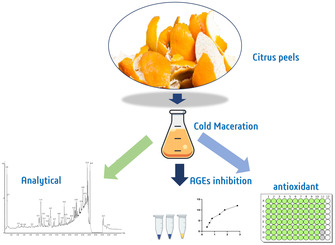

## 1 Introduction

Diabetes mellitus (DM) is metabolic disorders characterized by hyperglycemia, that may occur due to complete or partial deficiency of insulin (Egan and Dinneen 2018). DM can be classified into three major classes Type 1, Type 2 and Gestational diabetes (Ke et al. 2022). An uncontrolled and persistent hyperglycemia may facilitate glycation of proteins (Park et al. 2019). A more complicated form of these non‐enzymatic glycation reactions (Maillards reaction) between nucleic acids, lipids, and proteins is called AGEs (advanced glycated end products), that are infact a heterogeneous group of chemical moieties (Pérez‐Burillo, Rufián‐Henares, and Pastoriza 2019). AGEs are very crucial in progression of diabetic complications (Khalid, Petroianu, and Adem 2022). These complicated structural entities (AGEs) are mainly responsible for greatly effect protein functions by spontaneous modifications and thus stop working of specific features (Singh et al. 2014). The AGEs affect both long lived (elastin, collagen) and short‐lived proteins (plasma proteins) and thus lead to development of diabetic complications including retinopathy, nephropathy, neuropathy, neurological disorders (Parkinson's and Alzheimer's diseases) and several cardiovascular diseases (atherosclerosis, hypertension; Cole and Florez 2020). The AGEs mainly interact with RAGEs (receptor for AGEs) and increase inflammation and oxidative stress by activating NF‐κB, TGF‐β, MAPK/ERK pathways (Peng et al. 2016), which finally provoke epigenetic changes on longer basis (Perrone et al. 2020).

The AGEs also interact with DNA and aminophospholipids (Baynes 2000), that mainly occurs due to production of quiet diverse forms of AGEs including CML (*N*‐carboxymethyllysine) pentosidine, crossline, glyoxal‐lysine dimer (GOLD), methylglyoxal‐lysine dimer (MOLD), Glyxoyl (GO) and Methyl glyxoyl (MGO) (Gkogkolou and Böhm 2012). These last stage AGEs are formed in an irreversible reactions and act as intra‐ or intermolecular heterocyclic cross‐linker that leads to fragmentation in protein molecules (Jahan and Choudhary 2015). It is obvious that for the management of diabetes, the drug candidate with the capacity to manage glycemic index and stop or slow down AGEs formation is favored. Various compound classes are commonly being prescribed by physicians to manage blood glucose that include GLP1 receptor agonists, Thiazolidinediones, Dipeptidyl peptidase 4 inhibitors, Sulfonylureas, α‐Glucosidase inhibitors, Metformin (Ke et al. 2022). However, they have little or no effect on AGEs, and are provided with side effects including drug resistance, Dyslipidemia, GIT disturbances, hypoglycemia, dizziness (2%–7%), nervousness (4%), Edema and insomnia (Bytzer et al. 2001; Lorenzati et al. 2010).

Herbal medications have long been suggested for the treatment of diabetes (Ota and Ulrih 2017) due to safety, efficacy, and cost effectiveness (Kasole et al. 2019). *Galega officinalis* (Fabaceae), was the first medicinal plant with a definite antidiabetic action, and it has been used to treat diabetes mellitus since the Middle Ages (Hachkova et al. 2021). Galegine, a guanidine derivative, was isolated from this plant, also known as Italian fitch, French lilac, or goat's rue. This chemical compound possesses an identical chemical structure to that of metformin (anti‐diabetic drug) and the extract of this plant is responsible for reducing blood glucose levels (Ríos, Francini, and Schinella 2015).

Several phenolic substances, such as anthocyanins and flavonoids, have anti‐diabetic properties (Praparatana et al. 2022), that are mainly attributed to their strong antioxidant potential (Sarian et al. 2017). Further dietary flavonoids and polyphenolic compounds have applications in diabetes due to effects on lipid peroxidation, glucose absorption by GLUT2, and the inhibition of insulin dependent PI3K, activation of AMPK (Stewart et al. 2009; Alam, Meerza, and Naseem 2014) and decreased the genetic expression of PPARγ (Stanley Mainzen Prince and Kamalakkannan 2006). Likewise in case of AGEs inhibition, flavonoids may lessen diabetic nephropathy in NRK‐52E and RPTEC cells via suppressing RhoA/Rho‐kinase mediated pro‐inflammatory signaling (i.e., TNF‐α, IL‐1β, and TGF‐β1; Sharma et al. 2019).

*Citrus* is a broad genus of plants that has 156 species (Tanaka's classification). The grapefruits (*Citrus paradesi*) are commonly cultivated in several parts of world (Maqbool et al. 2023). Citrus peel extracts are a rich source of several types of flavonoids, flavonols, flavanones, isoflavones, and anthocyanidins and their derivatives (Duan et al. 2017; Li et al. 2022).

Citrus is a major fruit that shares a major part in exports from Pakistan. Despite its economic importance, little detail is available regarding the phytochemical and biological evaluation of *Citrus paradesi* variety from Pakistan especially in the context of AGEs. The study outcomes are valuable in using *Citrus paradesi* peel in herbal medicine.

## 2 Material and Methodology

### 2.1 Plant Collection, Identification, and Extraction

The fruits of *Citrus paradesi* were collected from local herbal market and authenticated at Islamabad Herberium, Quaid I Azam University Islamabad. Peels were removed and dried using ambient oven at 40°C. Afterward, dried plant material was powdered and stored at 2°C till further processing. The powdered plant material (200 g) was extracted using cold maceration for 7 days (three times) followed by solvent evaporation using rotary evaporator. Further liquid–liquid fractionation was performed to obtain extracts of diverse polarity.

### 2.2 Chemicals and Solvents

The chemicals used in the project including DPPH (2,2‐diphenyl‐1‐picrylhydrazyl), α‐glucosidase, Trolox, ABTS (2, 2′‐azino‐bis (3‐ethylbenzothiazoline‐6‐sulphonic acid)), HCL, Methyl Glyoxyl (MGO), H_2_folin–Ciocalteu's reagent, gallic acid, quercetin, TPTZ (2,4,6‐tripyridyl‐s‐triazine), FeCl_3_, aluminum chloride, NaH_2_PO_4_, Na_2_HPO_4_, Streptozotocin (STZ), (*p‐*nitrophenyl‐α‐D‐glucopyranoside, α‐glucosidase) ( *Saccharomyces cerevisiae* ) were purchased from Merck (UK), Oxoid (UK), Sigma Aldrich (USA). Glucometer and blood glucose test strips were purchased from Roche(UK). All solvents used during investigation will be HPLC grade including methanol, ethanol, dichloromethane, *n‐*hexane, ethyl acetate, and water were analytical grade (Acros, Geel, Belgium).

### 2.3 Phytochemical Screening

The quantitative phytochemical analysis was performed by using standard procedures (Ayoola et al. 2008) with slight modifications.

### 2.4 ATR‐FTIR Analysis

ATR‐FTIR analysis was performed to see the diversity of functional groups present in each citrus extract. FTIR spectrum was measured at range of 4000–400 cm^−1^ at 4 cm^−1^ intervals of spectral resolution (average 128 scans).

### 2.5 Chromatographic Analysis

#### 2.5.1 HPLC‐DAD Analysis

The HPLC analysis of tested samples was performed by using Agilent HPLC‐DAD system (Agilent 1200 series system, Agilent Technologies, Santa Clara, CA, USA). The sample (10 μL) was injected with a flow rate of 1 mL/min using water: acetonitrile (5%–100% acetonitrile) gradient system acidified with 0.1% formic acid. All analyses were performed on a Luna C18 column (silica, 250 × 4.6 mm, 5 μm; Phenomenex, Torrence, CA, USA).

#### 2.5.2 HPLC‐DAD‐QToF Analysis

A QToF spectrometer (Xevo, G2‐XS QToF spectrometer, Waters, Milford, MA, USA) together with an LC system (Acquity) was used for MS^2^ analysis of peel extracts by our previously reported method (Rafey et al. 2021). Briefly, 5 μL of the test sample was injected with a flow rate of 0.6 mL/min. Water:acetonitrile gradient mobile system, acidified with 0.1% formic acid was used during analysis for 40 min. The separation was achieved on a reverse phase column (BEH Shield, 1.7 μm, 100 × 2.10 mm, Waters, Milford, MA, USA). During first scan, a complete scan analysis (ESI [+] and ESI [−], m/z 50–1500) was recorded using sensitivity mode (approximate resolution: 22,000 FWHM), spray voltage was +1.0 kV and −0.8 kV, cone gas flow 50.0 L/h, with desolvation temperature at 120°C and 550°C, and desolvation gas flow 1000.0 L/h. The DAD spectral range was 190–500 nm. A standard mix comprising diverse flavonoids and polyphenolic compounds was also used.

#### 2.5.3 Total Phenolic (TPC) and Flavonoid Content (TFC)

The TPC and TFC of tested samples were determined using standard procedure (Stankovic 2011).

### 2.6 Antioxidant Activity

#### 2.6.1 DPPH (1, 1‐ Diphenyl‐2‐Picryl Hydrazyl) Assay

DPPH activity was determined by a modified method (Hussen and Endalew 2023). Briefly, 50 μL of DPPH (0.1 mM in methanol) reacted with 50 μL test material (0.93–120 μg/mL) and placed in dark place for 30 min. Afterward, the absorbance of reaction mixture was recorded at 517 nm on UV–Vis Spectrophotometer. Quercetin (0.4–60 μM) was used as reference standard.

#### 2.6.2 FRAP Assay

The FRAP assays (ferric‐reducing antioxidant power) was used as described previously. In brief, FRAP working mixture was prepared (TPTZ 10, 20 mM ferric chloride and 300 mM acetate buffer) and reacted with 20 μL of plant extract in a 96‐micrplate and incubated at room temperature (30 min) in dark place. Afterwards the absorbance was recorded at 560 nm.

### 2.7 Antidiabetic Assays

#### 2.7.1 α‐Glucosidase Inhibition Assay

The antidiabetic activity of test samples was accomplished by using with α‐glucosidase inhibition assay as described previously (Mahnashi et al. 2022). The test sample (1.5–0.01 mg/mL) was reacted with α‐glucosidase (0.2 units/mL in phosphate buffer, pH 6.8) and incubated for 15 min. Afterward, the 0.29 mM substrate (p‐nitrophenyl‐α‐D‐glucopyranoside) was added to this mixture and incubated for further 20 min. The Na_2_CO_3_ (100 μL, 200 mM) was used to terminate the reaction. Finally, the absorbance was measured at 400 nm. Acarbose (0.9–0.001 mg/mL) was used as standard drug. The % inhibition was recorded by following formula;

(1) $$\%\text{Inhibition}=\left[1-A_{1}/A_{2}\right]\times 100$$

where *A*_1_ is the absorbance of sample and *A*_2_ is the absorbance of control.

#### 2.7.2 AGEs Assay (Non‐oxidative Mode)

The non‐oxidative mode of AGEs formation was performed by BSA‐glucose glycation method (bovine serum albumin‐glucose) (Starowicz and Zieliński 2019). The reaction mixture contained BSA (10 mg/mL; 135 μL), test sample 30 μL (1–0.001 mg/mL), glucose (500 mM, 135 μL in phosphate buffer; 50 mM, pH 7.4) and Sodium Azide (0.02%) and incubated for 21 days at 37°C. The fraction unbound was removed by using TCA (trichloroacetic acid, 100%, 10 μL) and centrifuged. The pellet was dissolved in phosphate buffer saline (pH 10). Fluorescence intensity (ex 370/emis 440; ex 335/emis 385 nm) was recorded on a spectrofluorometer (FLx800, BioTek, Winooski, USA). Rutin (1–0.001 mg/mL) was used as standard drug. The % inhibition was calculated as per following formula;

(2) $$\%\text{Inhibition}=\left[1–F_{1}/F_{2}\right]\times 100$$

where *F*_1_ is the absorbance of sample and *F*_2_ is the absorbance of control. The IC_50_ was calculated by linear scatter plot.

#### 2.7.3 AGEs Assay (Oxidative Mode)

The oxidative mode of AGEs formation was accomplished by BSA‐MGO glycation method (bovine serum albumin‐methyl glyoxyl; Starowicz and Zieliński, 2019). Briefly, the BSA (10 mg/mL; 135 μL) and test sample (30 μL; 1–0.001 mg/mL) were added to MGO (5.75 mM, 135 μL using phosphate buffer; 50 mM, pH 7.4) and sodium azide (0.02%) and incubated for 24 h at 37°C. The change in fluorescence intensity (ex 335/emis 385 nm; ex 370/emis 440) was determined on a spectrofluorometer (FLUOstar Omega, BMG Lab Tech, Aylesbury, UK). Rutin (30 μL; 1–0.001 mg/mL) was used as standard drug:

(3) $$\%\text{Inhibition}=\left[1-F_{1}/F_{2}\right]\times 100$$

where *F*_1_ is the absorbance of sample and *F*_2_ is the absorbance of control. The IC_50_ was calculated by linear scatter plot.

### 2.8 Statistical Analysis

All biological activity experiments were performed in three independent experiments data was expressed as ±SD. One‐way ANOVA (followed by post hoc Tukey test) with *p* < 0.05 was used.

## 3 Results

### 3.1 Phytochemical Screening

After successive fractionation, prior quantitative phytochemical analysis was performed. As expected, the flavonoids were most prevalent compound class present in *Citrus paradisi* (Table 1).

**TABLE 1:** Quantitative photochemical analysis of *Citrus paradisi*.

Plant part	Type of compounds				
	Flavonoids	Steroids/triterpenoids	Alkaloids	Tannins	Saponins
MeOH Fract	++	++	−	−	−
Chloroform Fract	+++	++	−	−	−
Ethyl acetate Fract	+++	++	−	−	−
Aqueous Fract	++	++	−	+	−

*Note:* Phytochemicals: ++++ intensely present, ++ reasonably present, + marginally present, − negative.

Abbreviation: Fract, fraction.

### 3.2 FTIR Analysis

**FIGURE 1:**
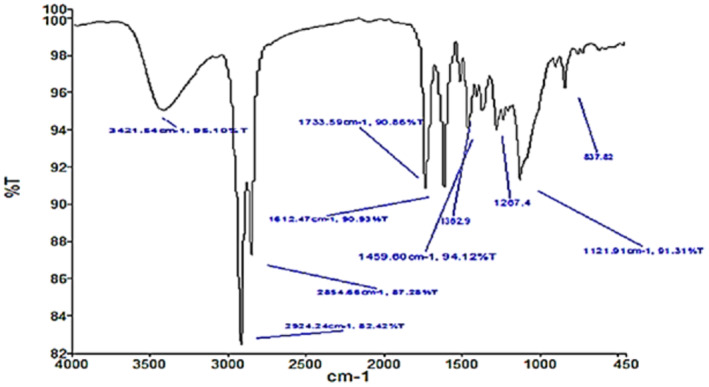
FTIR analysis of total *Citrus paradisi* extract.

The FTIR absorbance bands were recorded within the range of 400–4000 cm^−1^. Most prominent peaks were recorded at 3421.5, 2924.2, 2554.6, 1733.5, 1612.47, 1362.9 and 1121.9 cm^−1^ (Figure 1; Table 2). Carefully spectral comparison revealed the presence of functional groups pertaining to proteins, phenols, flavonoids, and methylated groups (Table 2).

**TABLE 2:** ATR‐FTIR analysis of *Citrus paradisi* extract.

Functional groups	Peak range (cm^−1^)	Bond	*Citrus paradisi*
Aliphatic amine	3300–3500	N‐H stretching	
Alcohol/phenol	3200–3600	O‐H stretching	3421.54
Alkanes	2850–2970	C‐H stretching	2924.24
	2850–2970	C‐H stretching	2854.66
Carbonyl	1690–1760	C‐H stretching	1733.59
Alkenes	1610–1680	‐C=C‐ stretching	1612.47
Alkyles			
R‐C(O)‐NH2	1590–1655	‐N‐H bending	
R‐C(O)‐NH‐R	1510–1560	N‐H bending	
Alkanes	1340–1470	C‐H Bending	1459.6
Ester (aromatic)	1365 + 1395	‐CH(CH3)_2_ Bending	1362.9
	1250–1310	O=C‐O‐C stretching	1267.4
Alcohol/carboxylic acid/ester/ether	1180–1360	C‐O Stretching	1121.9
Aliphatic amines	1050–1300	C‐O Stretching	
Alcohol/carboxylic acid/ester	1050–1300	C‐O	
Aromatic amine	1020–1250	C‐N Stretching	
Carboxylic	910–950	O‐H bending	837.4
Aliphatic bromo	500–600		

### 3.3 HPLC Analysis

**FIGURE 2:**
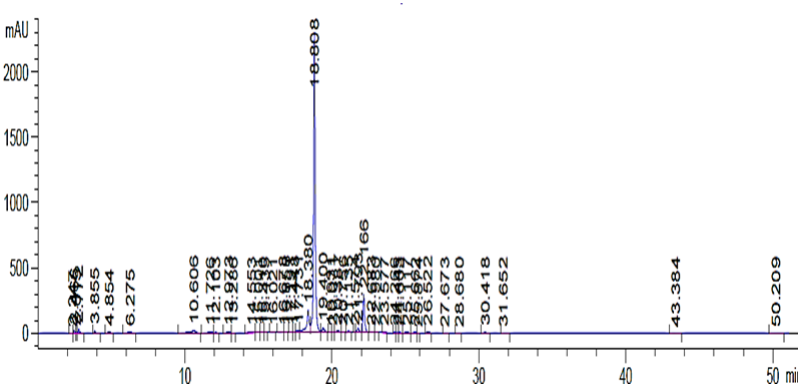
HPLC‐DAD chromatogram of *Citrus paradisi* chloroform fraction.

**FIGURE 3:**
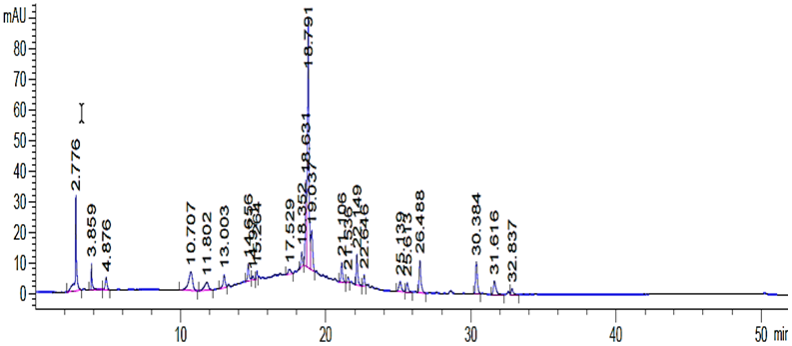
HPLC‐DAD chromatogram of *Citrus paradisi* (CHK) ethyl acetate fraction.

The chloroform fraction was analyzed and a less intense chromatogram was observed. A major peak at Rt 18.7 nm was observed that corresponded to the same compounds as seen in case of ethyl acetate fraction (Figure 2) with a less intensity. The ethyl acetate fraction of *C. paradisi* was analyzed for the presence of flavonoids and we were able to observe one major peak at Rt 18.80 min (> 2000 mAU at 280 nm; Figure 3).

### 3.4 LC–MS QTOF Analysis

**FIGURE 4:**
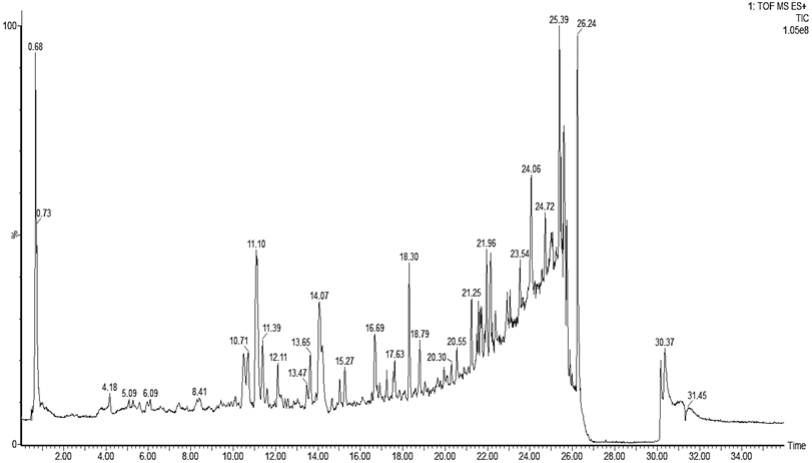
LC–MS QTOF (M^+H^) analysis of chloroform fraction of *Citrus paradisi*.

**FIGURE 5:**
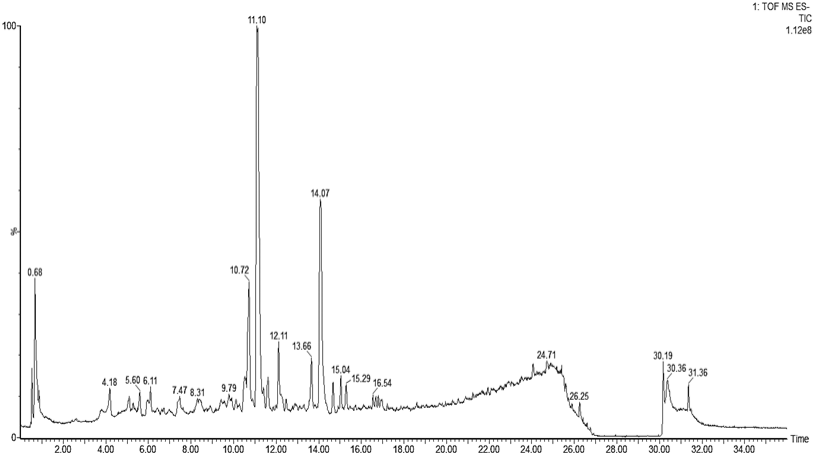
LC–MS QTOF (M^−H^) analysis of chloroform fraction of *Citrus paradisi*.

The LCMS‐QTOF analysis of peel extract of *C. paradesi* presented a complex data and the most prominent peak was identified was detected at Rt 11.10 min. Detailed mass fragment analysis comparison fragmentation, MS and MS^2^ spectra of this peak, and comparison with literature, it was identified as Naringin. The other identified compounds included Narirutin 4‐O‐glucoside (Rt 11.39) Naringenin‐7‐O‐rutinoside (Rt 10.72 min), and Nobilitein (Rt 17.6 min) and a series of polymethoxylated flavonoids (Figures 4 and 5, Table 3).

**TABLE 3:** MS and MS^2^ fragmentation of compounds detected in chloroform fraction.

Peak no	Compound identification	Formula	RT	[M‐H]^−^ (m/z)	[M‐H]^+^ (m/z)	MS/MS fragment signals (m/z)
1	Unidentified PMF		10.49		497.362	463.231, 277.123, 261.124; 189.6123;
	Naringenin‐7‐O‐rutinoside		10.72	579.6231		271.1254 (Roowi and Crozier 2011)
2	Naringin	C_27_H_32_O_14_	11.10		581.1930	435.1215; 419.1356; 383.1278; 339.1245, 315.1459; 273.3651; 195.0349, 153.1245; 129.2360; 85.3621 (Wang et al. 2019)
3	Narirutin 4‐O‐glucoside	C_33_H_42_O_19_	11.39	741.1245		579.1234 (Ali et al. 2022)
4	Hesperidin	C_28_H_34_O_15_	11.59		611.1973	303.1236; 465.1448 (Bouhafsoun et al. 2018)
	Rhoifolin	C_27_H_30_O_14_	12.11		579.1782	433.2561; 271.0645 (Ali et al. 2022)
5	Unidentified PMF	C_27_H_28_O_15_	13.47		581.1962	419.1366; 331.1152; 291.1237; 219.074
6	Luteolin‐7‐*O*rutinoside	C_27_H_30_O_16_	13.65	593.1241		285.3214 (Roowi and Crozier, 2011)
7	Poncirin	C_28_H_34_O_14_	14.07		595.1473	449.1235; 344.0236; 287.1243;161.05 75 (Luo et al. 2019)
8.	Unidentified PMF	C_31_H_51_O_9_	14.20		589.3346	567.3506; 433.1542; 287.1522
9.	Unidentified PMF	C_20_H_37_O_15_	15.02		517.2314	495.2214; 333.1677; 297.1423; 279.0123; 214.08891; 158.0156
10.	Isonaringin	C_27_H_32_O_14_	15.27		581.0124	419.1234;211.05821;149.0228; 121.06 26 (Brits et al. 2021)
11.	Unidentified PMF	C_20_H_21_O_7_	16.69		373.1239	355.1565; 297.1520; 214.0912; 163.0421
12.	Hexamethoxyflavone	C_21_H_22_O_8_	17.26		403.3654	373.0216; 388.1143; 355.0123; 135.0243 (Brits et al. 2021)
13.	Sinensitin	C_20_H_20_O_7_	17.56		373.2614	343.2145; 357.3654; 358.1234;312.9824 (Brits et al. 2021)
14.	Nobilitin	C_21_H_22_O_8_	17.63		403.2145	388.1142; 373.093; 355.0945 (Brits et al. 2021)
15.	5,6,7,3’,4’,5’‐Hexamethoxyflavone	C_21_H_22_O_8_	18.30		403.1408	373.0817; 355.1207; 388.1020 (Brits et al. 2021)
16.	3,5,6,7,8,3’,4’ Heptamethoxyflavone	C_22_H_24_O_9_	18.79		433.2314	403.1134; 418.1147; 385.0614 388.0236; 417.1178 (Brits et al. 2021)
17.	Hexamthoxyflavone	C_21_H_22_O_8_	20.3		373.0917	373.0917, 388.1233; 355.1237 135.0623 (Brits et al. 2021)

Abbreviation: PMF, polymethoxylated flavonoid.

### 3.5 Biological Activities

#### 3.5.1 Total Phenolics, Total Flavonoids, and Antioxidant Activities

In case of *Citrus paradesi*, high phenolic contents were observed in total extract (50.2 ± 0.2 mg/gGAE). Amongst fractions, ethyl acetate (75.2 ± 0.34 mg/gGAE) and chloroform fractions (62.3 ± 0.41 mg/gGAE) had highest phenolic contents (Table 4). The total flavonoids contents (TFC) were high comparably high in ethyl acetate (25.6 ± 0.12 mg/gRUE) and chloroform (20.4 ± 0.19 mg/gRUE; Table 4).

**TABLE 4:** Quantitative determination of phenolics, flavonoids and antioxidant profile of *citrus paradesi*.

Sample	TPC (mg/gGAE^a^)	TFC (mg/gRUE^b^)	DPPH (IC_50_ μg/mL)	FRAP (μg/g of FeSO_4_)
Total extract	50.2 ± 0.20	19.3 ± 0.26	53.2 ± 0.10	120.2 ± 0.70
Ethyl acetate Fract	75.2 ± 0.34	25.6 ± 0.12	20.3 ± 0.22	85 ± 0.23
Chloroform Fract	62.3 ± 0.41	20.0 ± 0.19	15.3 ± 0.11	65 ± 0.19
Reference standard			6.27 ± 0.02^c^	

Abbreviations: DPPH, 2,2‐diphenyl‐1‐picrylhydrazyl; Fract, fraction; FRAP, ferrous reducing antioxidant power; TFC, total flavonoid contents; TPC, total phenolic contents.

^a^ mg equivalents per gram of gallic acid.

^b^ mg equivalents per gram of rutin.

^c^ Quercetin (μM).

The *C. paradesi* fractions were analyzed for DPPH radical scavenging and FRAP assays (Table 4). The DPPH assay revealed a strong radical activity of total crude extract (IC_50_ 53.2 ± 0.10 μg/mL). Amongst individual fractions, chloroform (IC_50_ 15.3 ± 0.11 μg/mL) and ethyl acetate fractions (IC_50_ 20.3 ± 0.22 μg/mL) presented significant inhibition. Likewise, high FRAP value was recorded by total extract of *C. paradesi* (120.2 ± 0.70 μg/g of FeSO_4_). In comparison with total extracts, highest FRAP was reported by ethyl acetate (85 ± 0.23 μg/g of FeSO_4_) and chloroform fractions (85 ± 0.23 μg/g of FeSO_4_; Table 4).

#### 3.5.2 Antidiabetic and Antiglycation Assays

*Citrus paradesi* extract presented a moderate inhibition of α‐glucosidase (IC_50_ 1.23 mg/mL), whereas a comparably high activity was noticed in chloroform fraction (IC_50_ 0.69 mg/mL) and ethyl acetate fraction was in‐active (Table 5). In AGEs inhibition assays, both non‐oxidative and oxidative models were used. In non‐oxidative model (BSA‐glucose), total extract was able to show moderate inhibition of AGEs (IC_50_ 0.63 mg/mL), while a significant activity was recorded in case of chloroform fraction (IC_50_ 0.23 mg/mL; Table 5). Conversely, in case of BSA‐MGO model, a promising inhibition (IC_50_ 0.5 mg/mL) was observed in case of total extract, whereas significant inhibition was recorded in case of chloroform fraction (IC_50_ 0.1 mg/mL) and ethyl acetate fractions (IC_50_ 0.78 mg/mL; Table 5).

**TABLE 5:** AGEs and α‐glucosidase assay of *Citrus paradesi*.

Sample	BSA‐Glc (IC_50_ mg/mL)	BSA‐MGO (IC_50_ mg/mL)	α‐glucosidase (IC_50_ mg/mL)
Total extract	0.63	0.5	1.23
Chloroform Fract	0.23	0.10	0.69
Ethyl acetate Fract	Inactive	0.78	Inactive
*Standard*	0.04^a^	0.84^a^	0.01^b^

Abbreviations: BSA‐Glc, bovine serum albumin‐glucose; BSA‐MGO, bovine serum albumin‐methyl glyoxyl; Fract, fraction.

^a^ Rutin.

^b^ Acarbose.

## 4 Discussion

The citrus peels have long been used for ailments of various health‐related problems, especially diabetes, digestive disorders, cancer, atherosclerosis, and inflammation (Rafiq et al. 2018; Maeda‐Yamamoto et al. 1999). In our project, *C. paradesi* (Pakistan origin) was investigated for its antidiabetic and antiglycation potential. Preliminary phytochemical analysis revealed a significant presence of flavonoids and triterpenoids in all fractions. Quantitative phytochemical analysis revealed the presence of high phenolic contents compared to flavonoid contents as reported earlier (Ghasemi, Ghasemi, and Ebrahimzadeh 2009). Further variation in quantification results can be noticed since several factors can affect detection values for instance extraction methods, degree of polymerization of phenolics and flavonoids, seasonal variations, and solvent polarity (Ștefănescu et al. 2019). The citrus peels are chief source of diverse flavonoids especially polymethoxylated flavones (PMF), C‐ or O‐glycosylated flavones, O‐glycosylated flavanones, flavonols, and many other different phenolic acids, along with related derivatives (Anagnostopoulou et al. 2005). It has been reported by several investigations that the majority of these activities are attributed to strong antioxidant activities (Goulas, Tzortzis, and Gibson 2007). The presence of phenolic compounds was further confirmed by using ATR‐FTIR analysis. The signal ranges were compared with literature and standard FTIR shift data (Tayyab et al. 2021). A broad peak was recorded at 3421.5 cm^−1^ indicating the existence of –OH group, while the bands centered at 1733.5 and 1612.4 cm^−1^ were assigned to the C=O group and C=C stretching. The band seen at 2924 and 2854 cm^−1^ can be attributed to the C–H stretching vibrations of methyl, methylene, and methoxy groups as reported earlier (Kumar et al. 2014). The peak at 1362 cm^−1^ was assigned to the aromatic C=C bond (Sangeetha et al. 2011), whereas peak at 1459.6 was assigned to C‐H Bending vibrations. The band at 837.4 cm^−1^ signifies presence of R–CH group. The signals from OH and carboxylic acid may refer to diverse flavonoids (Yee et al. 2004).

The HPLC profiling of *Citrus paradisi* was performed and a series of compounds with diverse polarity were observed. The chloroform fraction was analyzed on HPLC‐DAD and a complex chromatogram was recorded. The initial peaks ranging from Rt 3.85 to 6.27 min were less intense and were considered as representing less polar compounds. Whereas comparably larger peaks were recorded at 10.66, 13.28, and 18.38 min. A sharp peak at Rt 18.7 min (> 2000 mAU) was observed that represented a major compound in the fraction. Similarly, significant peaks were recorded at Rt 19.4 min and Rt 22.031 min. The ethyl acetate fraction of *Citrus paradisi* also presented peaks at similar retention time with lower intensities, thus for further analytical analysis it was decided to carry investigations on chloroform fraction. Upon comparison of HPLC analysis with previously published data, it was evident that both medium polar fractions (chloroform and ethyl acetate) could be flavonoid glycosides (Hung et al. 2011). Numerous peaks with comparably less intensity may possibly correspond to polymethoxylated flavonoids (Zhang et al. 2012), including naringin and hesperidin, since they are major flavonoids reported earlier in *Citrus paradisi* (Ahmed et al. 2019). Smaller peaks may correspond to a series of flavonoids and polyphenolic compounds including Gallic acid, nobiletin tangeritin narirutin, and sinensetin as reported earlier (Ko et al. 2010).

The Ms‐Ms^2^ analysis of *C. paradesi* chloroform fraction showed a series of compounds representing several flavonoid classes including polymethoxylated flavonoids, flavones, flavanones, flavonols, and flavonoid glycosides (Figures 4 and 5). Most commonly reported flavonoids in *Citrus* spp. including hesperidin, naringin, Sinensitin, Nobilitin, and naringenin (Khan, Huma, and Dangles 2014), were noticed in *C. paradesi*. These flavonoids are reported with strong antioxidant activities (Horbańczuk et al. 2019). Previous investigations have proved that strong antioxidant activities of flavonoid molecules are mainly linked with “OH” group substitutions at “B” ring in flavonoid skeleton (Khlebnikov et al. 2007; Masek et al. 2017).

The polymethoxylated flavonoids (PMF) are characterized by the presence of more than two OCH_3_ groups on benzo‐γ‐pyrone (15‐carbon, C6, C3, and C6) skeleton with a carbonylic moiety on C4 (Gao et al. 2018). The PMFs compared to flavonoids with OH groups are more bioavailable (high membrane permeability) due to polar nature (Benaventegarcía and Castillo 2008). The PMFs are therefore more biological active (Walle 2007).

Strong antioxidant activities of medicinal plants are mainly attributed to high flavonoid and phenolic contents (Catherine, Rice‐Evans, Nicholas, and George 1996), and based on LCMS‐QToF analysis, since *C. paradesi* chloroform fraction was found rich in diverse flavonoids, antioxidant potential was determined. It was evident that *C. paradesi* extracts possessed significant antioxidant activities that were due to high phenolic and flavonoid contents (Table 4). Antioxidant feature of flavonoids and other compounds make them effective for ailment of various health issues and most of biological activities since reactive oxygen species (ROS)‐induced oxidation may provoke cell‐membrane disintegration, damage of membrane proteins, and DNA damage. All these may lead to development of diabetes, cancer, cardiovascular pathologies, and neurodegenerative issues (Ramful et al. 2015). We further analsyed *C. paradesi* fractions for antidiabetic and advanced glycation inhibitory potential. Total crude extract of *C. paradesi* inhibited both α‐glucosidase and Advance glycation End products both in oxidative and non‐oxidative modes. This was mainly contributed by chloroform fraction that showed significant antidiabetic and AGEs inhibitory properties. As observed earlier since this fraction was rich in flavonoids especially polymethoxylated flavonoids, they may have a major contribution in this case. Earlier correlation studies have confirmed that a positive correlation exists between AGEs and antioxidant properties of plant extracts (Upadhyay et al. 2014). Further, it has been well established that this activity could possibly be due to preventive effect in dicarbonyl formation (Thornalley, Langborg, and Minhas 1999). The α‐glucosidase inhibitory potential of flavonoids is mainly due presence of the OH group at C‐4′ and double bond at C‐2 and C‐3 (Şöhretoğlu and Sari 2020). The OH groups are crucial for enzymatic binding and similarly flavonoids with methoxy groups especially at position number 7 are more good OCH_3_ α‐glucosidase inhibitors (Potipiranun et al. 2018).

## 5 Conclusion

*Citrus* peel is a rich source of diverse flavonoids and polyphenolic compounds including polymethoxylated flavonoids which are reported with several biological activities. It was concluded that medium polar fractions of *Citrus paradisi* peel extracts possess potential antidiabetic and ant AGEs potentials, that is mainly attributed to presence of flavonoids and especially the polymethoxylated flavonoids. Thus, medium polar fraction of *Citrus paradisi* can be used as an effective treatment option for diabetics mellitus and associated health concerns. Keeping in mind the importance of these findings, further investigations can be performed to quantify major flavonoids, their individual bioactivities and in vivo investigations. Formulation design studies are proposed on quantified extracts.

## Author Contributions

**Adnan Amin:** conceptualization (equal), data curation (equal), supervision (equal), validation (equal), writing – review and editing (equal). **Muhammad Fakhar Ul Mehmood:** data curation (lead), formal analysis (lead), software (supporting). **Marvi:** formal analysis (supporting), investigation (supporting), project administration (supporting), resources (lead). **Hatem A. Abuelizz:** formal analysis (lead), funding acquisition (supporting), investigation (supporting), project administration (supporting), resources (equal). **Nighat Aziz:** software (equal), writing – review and editing (equal). **Raheela Bano:** funding acquisition (supporting), resources (supporting), visualization (lead). **Imran Ahmad:** software (lead), visualization (supporting). **Asif Wazir:** software (equal), validation (equal). **Saiqa Ishtiaq:** resources (lead), software (supporting), supervision (supporting).

## Conflicts of Interest

The authors declare no conflicts of interest.

## Data Availability

The authors have nothing to report.

## References

[fsn34602-bib-0001] Ahmed, W. , R. Azmat , A. Qayyum , et al. 2019. “Extraction of Diverse Polyphenols in Relation With Storage Periods of *Citrus paradisi* CV. Shamber Through HPLC‐DAD Technique Using Different Solvent.” Journal of Food Science and Technology 56: 384–390. 30728581 10.1007/s13197-018-3499-x PMC6342808

[fsn34602-bib-0002] Alam, M. M. , D. Meerza , and I. Naseem . 2014. “Protective Effect of Quercetin on Hyperglycemia, Oxidative Stress and DNA Damage in Alloxan Induced Type 2 Diabetic Mice.” Life Sciences 109: 8–14. 10.1016/j.lfs.2014.06.005. 24946265

[fsn34602-bib-0101] Ali, A. , J. J. Cottrell , and F. R. Dunshea . 2022. “LC‐MS/MS Characterization of Phenolic Metabolites and Their Antioxidant Activities from Australian Native Plants.” Metabolites 12: 1016. 10.3390/metabo12111016. 36355099 PMC9698446

[fsn34602-bib-0004] Anagnostopoulou, M. A. , P. Kefalas , E. Kokkalou , A. N. Assimopoulou , and V. P. Papageorgiou . 2005. “Analysis of Antioxidant Compounds in Sweet Orange Peel by HPLC‐Diode Array Detection‐Electrospray Ionization Mass Spectrometry.” Biomedical Chromatography 19: 138–148. 15515108 10.1002/bmc.430

[fsn34602-bib-0005] Ayoola, G. A. , H. B. Coker , S. A. Adesegun , et al. 2008. “Phytochemical Screening and Antioxidant Activities of Some Selected Medicinal Plants Used for Malaria Therapy in Southwestern Nigeria.” Tropical Journal of Pharmaceutical Research 7: 1019–1024.

[fsn34602-bib-0006] Baynes, J. W. 2000. “From Life to Death–The Struggle Between Chemistry and Biology During Aging: The Maillard Reaction as an Amplifier of Genomic Damage.” Biogerontology 1: 235–246. 11707900 10.1023/a:1010034213093

[fsn34602-bib-0007] Benaventegarcía, O. , and J. Castillo . 2008. “Update on Uses and Properties of Citrus Flavonoids: New Findings in Anticancer, Cardiovascular, and Anti‐Inflammatory Activity.” Journal of Agriculture and Food Chemistry 56, no. 15: 6185–6205. 10.1021/jf8006568 18593176

[fsn34602-bib-0104] Bouhafsoun, A. , M. A. Yilmaz , A. Boukeloua , H. Temel , and M. K. Harche . 2018. “Simultaneous Quantification of Phenolic Acids and Flavonoids in *Chamaerops humilis* L. Using LC–ESI‐MS/MS.” Food Science & Technology, Campinas 38, no. Suppl. 1: 242–247.

[fsn34602-bib-0106] Brits, M. , T. Naessens , M. Theunis , et al. 2021. “Identification and Quantification of Polymethoxylated Flavonoids in Different Citrus Species Using UPLC‐QTOF‑MS/MS and HPLC‑DAD.” Planta Medica 87: 1080–1088. 10.1055/a-1551-6337. 34412145

[fsn34602-bib-0010] Bytzer, P. , N. J. Talley , M. P. Jones , and M. Horowitz . 2001. “Oral Hypoglycaemic Drugs and Gastrointestinal Symptoms in Diabetes Mellitus.” Aliment Pharmacological Therapy 15, no. 1: 137–142. 10.1046/j.1365-2036.2001.00896.x 11136287

[fsn34602-bib-0011] Catherine, A. , J. M. Rice‐Evans, Nicholas , and P. George . 1996. “Structure‐Antioxidant Activity Relationships of Flavonoids and Phenolic Acids.” Free Radical Biology and Medicine 20, no. 7: 933–956. 8743980 10.1016/0891-5849(95)02227-9

[fsn34602-bib-0012] Cole, J. B. , and J. C. Florez . 2020. “Genetics of Diabetes Mellitus and Diabetes Complications.” Nature Reviews Nephrology 16: 377–390. 32398868 10.1038/s41581-020-0278-5 PMC9639302

[fsn34602-bib-0013] Duan, L. , L. L. Dou , K. Y. Yu , et al. 2017. “Polymethoxyflavones in Peel of *Citrus reticulata* ‘Chachi’ and Their Biological Activities.” Food Chemistry 1, no. 234: 254–261. 10.1016/j.foodchem.2017.05.018 28551233

[fsn34602-bib-0014] Egan, A. M. , and S. F. Dinneen . 2018. “What Is Diabetes? Key Points.” Medicine 10, no. 2: 1–4. 10.1016/j.mpmed.2018.10.002.

[fsn34602-bib-0015] Gao, Z. , W. Gao , S.‐L. Zeng , P. Li , and E. H. Liu . 2018. “Chemical Structures, Bioactivities and Molecular Mechanisms of Citrus Polymethoxyflavones.” Journal of Functional Foods 40: 498–509.

[fsn34602-bib-0016] Ghasemi, K. , Y. Ghasemi , and M. A. Ebrahimzadeh . 2009. “Antioxidant Activity, Phenol and Flavonoid Contents of 13 Citrus Species Peels and Tissues.” Pakistan Journal of Pharmaceutical Sciences 22, no. 3: 277–281. 19553174

[fsn34602-bib-0017] Gkogkolou, P. , and M. Böhm . 2012. “Advanced Glycation End Products: Key Players in Skin Aging?” Dermato‐Endocrinology 4, no. 3: 259–270. 23467327 10.4161/derm.22028 PMC3583887

[fsn34602-bib-0018] Goulas, A. , G. Tzortzis , and G. R. Gibson . 2007. “Development of a Process for the Production and Purification of *α*‐ and *β*‐Galactooligosaccharides From *Bifidobacterium bifidum* NCIMB 41171.” International Dairy Journal 17: 648–656.

[fsn34602-bib-0019] Hachkova, H. , M. Nagalievska , Z. Soliljak , et al. 2021. “Medicinal Plants *Galega officinalis* L. and Yacon Leaves as Potential Sources of Antidiabetic Drugs.” Antioxidants 10, no. 9: 1362. 34572994 10.3390/antiox10091362 PMC8466348

[fsn34602-bib-0020] Horbańczuk, O. K. , M. A. Kurek , A. G. Atanasov , M. Brnčić , and S. R. Brnčić . 2019. “The Effect of Natural Antioxidants on Quality and Shelf Life of Beef and Beef Products.” Food Technology and Biotechnology 57: 439–447. 32123506 10.17113/ftb.57.04.19.6267 PMC7029390

[fsn34602-bib-0022] Hung, T. M. , T. D. Cuong , N. H. Dang , et al. 2011. “Flavonoid Glycosides From *Chromolaena odorata* Leaves and Their In Vitro Cytotoxic Activity.” Chemical and Pharmaceutical Bulletin 59, no. 1: 129–131. 21212562 10.1248/cpb.59.129

[fsn34602-bib-0023] Hussen, E. M. , and S. A. Endalew . 2023. “In Vitro Antioxidant and Free‐Radical Scavenging Activities of Polar Leaf Extracts of Vernonia Amygdalina.” BMC Complementary Medicine and Therapy 23, no. 1: 146. 10.1186/s12906-023-03923-y PMC10157976 37143058

[fsn34602-bib-0024] Jahan, H. , and M. I. Choudhary . 2015. “Glycation, Carbonyl Stress and AGEs Inhibitors: A Patent Review.” Expert Opinion in Therapeutic Patents 25: 12671284. 10.1517/13543776.2015.1076394 26293545

[fsn34602-bib-0025] Kasole, R. , H. D. Martin , and K. Kimiywe . 2019. “Traditional Medicine and Its Role in the Management of Diabetes Mellitus: “Patients' and Herbalists' Perspectives”.” Evidence‐Based Complementary and Alternative Medicine 2019: 2835691. 31354852 10.1155/2019/2835691 PMC6637672

[fsn34602-bib-0026] Ke, C. , K. M. V. Narayan , J. C. N. Chan , P. Jha , and B. R. Shah . 2022. “Pathophysiology, Phenotypes and Management of Type 2 Diabetes Mellitus in Indian and Chinese Populations.” Nature Reviews. Endocrinology 18: 413–432. 10.1038/s41574-022-00669-4. PMC9067000 35508700

[fsn34602-bib-0027] Khalid, M. , G. Petroianu , and A. Adem . 2022. “Advanced Glycation End Products and Diabetes Mellitus: Mechanisms and Perspectives.” Biomolecules 12, no. 4: 542. 35454131 10.3390/biom12040542 PMC9030615

[fsn34602-bib-0028] Khan, M. K. , Z. E. Huma , and O. Dangles . 2014. “A Comprehensive Review on Flavanones, the Major Citrus Polyphenols.” Journal of Food Composition and Analysis 33: 85–104.

[fsn34602-bib-0029] Khlebnikov, A. I. , I. A. Schepetkin , N. G. Domina , L. N. Kirpotina , and M. T. Quinn . 2007. “Improved Quantitative Structure–Activity Relationship Models to Predict Antioxidant Activity of Flavonoids in Chemical, Enzymatic, and Cellular Systems.” Bioorganic and Medicinal Chemistry 15: 1749–1770. 17166721 10.1016/j.bmc.2006.11.037 PMC2013303

[fsn34602-bib-0030] Ko, H. C. , M. G. Jang , C. H. Kang , et al. 2010. “Preparation of a Polymethoxyflavone‐Rich Fraction (PRF) of Citrus Sunki Hort. Ex Tanaka and Its Antiproliferative Effects.” Food Chemistry 123: 484–488.

[fsn34602-bib-0031] Kumar, A. , Y. S. Negi , V. Choudhary , and N. K. Bhardwaj . 2014. “Characterization of Cellulose Nanocrystals Produced by Acid‐Hydrolysis From Sugarcane Bagasse as Agro‐Waste.” Journal of Materials Physics and Chemistry 2, no. 1: 1–8.

[fsn34602-bib-0032] Li, P. , X. Yao , Q. Zhou , X. Meng , T. Zhou , and Q. Gu . 2022. “Citrus Peel Flavonoid Extracts: Health‐Beneficial Bioactivities and Regulation of Intestinal Microecology *In Vitro* .” Frontiers in Nutrition 24, no. 9: 888745. 10.3389/fnut.2022.888745 PMC9171401 35685878

[fsn34602-bib-0033] Lorenzati, B. , C. Zucco , S. Miglietta , F. Lamberti , and G. Bruno . 2010. “Oral Hypoglycemic Drugs: Pathophysiological Basis of Their Mechanism of Action Oral Hypoglycemic Drugs: Pathophysiological Asis of Their Mechanism of Action.” Pharmaceuticals 3: 3005–3020. 10.3390/ph3093005. 27713388 PMC4034109

[fsn34602-bib-0105] Luo, Y. , W. Zeng , K. E. Huang , et al. 2019. “Discrimination of *Citrus reticulata* Blanco and *Citrus reticulata* ‘Chachi’ as Well as the *Citrus reticulata* ‘Chachi’ Within Different Storage Years Using Ultra High Performance Liquid Chromatography Quadrupole/Time‐of‐Flight Mass Spectrometry Based Metabolomics Approach.” Journal of Pharmaceutical and Biomedical Analysis 171: 218–231. 10.1016/j.jpba.2019.03.056. 31072532

[fsn34602-bib-0035] Maeda‐Yamamoto, M. , H. Kawahara , N. Tahara , K. Tsuji , Y. Hara , and M. Isemura . 1999. “Effect of Tea Polyphenols on the Invasion and Matrix Metalloproteinases Activities of Human Fibrosarcoma HT1080 Cells.” Journal of Agriculture and Food Chemistry 47: 2350–2354. 10.1021/jf9811525 10794635

[fsn34602-bib-0036] Mahnashi, M. H. , Y. S. Alqahtani , B. A. Alyami , et al. 2022. “HPLC‐DAD Phenolics Analysis, α‐Glucosidase, α‐Amylase Inhibitory, Molecular Docking and Nutritional Profiles of *Persicaria hydropiper* L.” BMC Complementary Medicine and Therapies 22: 26. 35086537 10.1186/s12906-022-03510-7 PMC8793238

[fsn34602-bib-0037] Maqbool, Z. , W. Khalid , H. T. Atiq , et al. 2023. “Citrus Waste as Source of Bioactive Compounds: Extraction and Utilization in Health and Food Industry.” Molecules 28, no. 4: 1636. 36838623 10.3390/molecules28041636 PMC9960763

[fsn34602-bib-0038] Masek, A. , E. Chrzescijanska , M. Latos , and M. Zaborski . 2017. “Influence of Hydroxyl Substitution on Flavanone Antioxidants Properties.” Food Chemistry 215: 501–507. 27542504 10.1016/j.foodchem.2016.07.183

[fsn34602-bib-0039] Ota, A. , and N. P. Ulrih . 2017. “An Overview of Herbal Products and Secondary Metabolites Used for Management of Type Two Diabetes.” Frontiers in Pharmacology 8: 436. 10.3389/FPHAR.2017.00436/BIBTEX. 28729836 PMC5499308

[fsn34602-bib-0040] Park, S. , H. J. Kang , J. H. Jeon , M. J. Kim , and I. K. Lee . 2019. “Recent Advances in the Pathogenesis of Microvascular Complications in Diabetes.” Archives of Pharmacal Research 42, no. 3: 252–262. 10.1007/s12272-019-01130-3. 30771210

[fsn34602-bib-0041] Peng, Y. , J. M. Kim , H. S. Park , et al. 2016. “AGE‐RAGE Signal Generates a Specific NF‐κB RelA "Barcode" That Directs Collagen I Expression.” Scientific Reports 6: 18822. 26729520 10.1038/srep18822 PMC4700418

[fsn34602-bib-0042] Pérez‐Burillo, S. , J. Á. Rufián‐Henares , and S. Pastoriza . 2019. “Effect of Home Cooking on the Antioxidant Capacity of Vegetables: Relationship With Maillard Reaction Indicators.” Food Research International 121: 514–523. 31108776 10.1016/j.foodres.2018.12.007

[fsn34602-bib-0043] Perrone, A. , A. Giovino , J. Benny , and F. Martinelli . 2020. “Advanced Glycation End Products (AGEs): Biochemistry, Signaling, Analytical Methods, and Epigenetic Effects.” Oxidative Medicine and Cellular Longevity 20, no. 6: 3818196–3818213. 10.1155/2020/3818196 PMC7104326 32256950

[fsn34602-bib-0044] Potipiranun, T. , S. Adisakwattana , W. Worawalai , R. Ramadhan , and P. Phuwapraisirisan . 2018. “Identification of Pinocembrin as an Anti‐Glycation Agent and α‐Glucosidase Inhibitor From Fingerroot ( *Boesenbergia rotunda* ): The Tentative Structure–Activity Relationship Towards mg‐Trapping Activity.” Molecules 23: 3365. 30572593 10.3390/molecules23123365 PMC6321453

[fsn34602-bib-0045] Praparatana, R. , P. Maliyam , L. R. Barrows , and P. Puttarak . 2022. “Flavonoids and Phenols, the Potential Anti‐Diabetic Compounds From *Bauhinia strychnifolia* Craib Stem.” Molecules 27, no. 8: 2393. 35458587 10.3390/molecules27082393 PMC9032570

[fsn34602-bib-0046] Rafey, A. , A. Amin , M. Kamran , et al. 2021. “Analysis of Plant Origin Antibiotics Against Oral Bacterial Infections Using in Vitro and in Silico Techniques and Characterization of Active Constituents.” Antibiotics 10: 1504. 10.3390/antibiotics10121504. 34943716 PMC8699006

[fsn34602-bib-0047] Rafiq, S. , R. Kaul , S. A. Sofi , N. Bashir , B. Nazir , and G. A. Nayik . 2018. “Citrus Peel as a Source of Functional Ingredient: A Review.” Journal of the Saudi Society of Agricultural Sciences 17, no. 4: 351–358.

[fsn34602-bib-0048] Ramful, D. , T. Bahorun , E. Bourdon , E. Tarnus , and O. I. Aruoma . 2015. “Bioactive Phenolics and Antioxidant Propensity of Flavedo Extracts of Mauritian Citrus Fruits: Potential Prophylactic Ingredients for Functional Foods Application.” Toxicology 278, no. 1: 75–87. 10.1016/j.tox.2010.01.012 20100535

[fsn34602-bib-0049] Ríos, J. L. , F. Francini , and G. R. Schinella . 2015. “Natural Products for the Treatment of Type 2 Diabetes Mellitus.” Planta Medica 81, no. 12/13: 975–994. 10.1055/S-0035-1546131. 26132858

[fsn34602-bib-0102] Roowi, S. , and A. Crozier . 2011. “Flavonoids in Tropical Citrus Species.” Journal of Agriculture & Food Chemistry 59, no. 22: 12217–12225. 10.1021/jf203022f 21978223

[fsn34602-bib-0071] Sangeetha, G. , S. Rajeshwari , and VenckateshV. 2011. “Green Synthesis of Zinc Oxide Nanoparticles by Aloe Barbadensis Miller Leaf Extract: Structure and Optical Properties.” Material Research Bulletin 46: 2560–2566.

[fsn34602-bib-0051] Sarian, M. N. , Q. U. Ahmed , S. Z. Mat So'ad , et al. 2017. “Antioxidant and Antidiabetic Effects of Flavonoids: A Structure‐Activity Relationship Based Study.” Biomed Research International 2017: 8386065. 29318154 10.1155/2017/8386065 PMC5727842

[fsn34602-bib-0052] Sharma, D. , P. Gondaliya , V. Tiwari , and K. Kalia . 2019. “Kaempferol Attenuates Diabetic Nephropathy by Inhibiting RhoA/Rho‐Kinase Mediated Inflammatory Signalling.” Biomedicine Pharmacotherapy 109: 1610–1619. 30551415 10.1016/j.biopha.2018.10.195

[fsn34602-bib-0053] Singh, V. P. , A. Bali , N. Singh , and A. S. Jaggi . 2014. “Advanced Glycation End Products and Diabetic Complications.” Korean Journal of Physiological Pharmacology 18, no. 5: 1–14. 10.4196/kjpp.2014.18.1.1 PMC3951818 24634591

[fsn34602-bib-0054] Şöhretoğlu, D. , and S. Sari . 2020. “Flavonoids as Alpha‐Glucosidase Inhibitors: Mechanistic Approaches Merged With Enzyme Kinetics and Molecular Modelling.” Phytochemsirty Reviwes 19: 1081–1092. 10.1007/s11101-019-09610-6.

[fsn34602-bib-0055] Stankovic, M. S. 2011. “Total Phenolic Content, Flavonoid Concentration and Antioxidant Activity of *Marrubium peregrinum* L. Extracts.” Kragujevac Journal of Science 33: 63–72.

[fsn34602-bib-0056] Stanley Mainzen Prince, P. , and N. Kamalakkannan . 2006. “Rutin Improves Glucose Homeostasis in Streptozotocin Diabetic Tissues by Altering Glycolytic and Gluconeogenic Enzymes.” Journal of Biochemistry Molecular Biology and Toxicology 20: 96–102. 10.1002/jbt.20117 16615078

[fsn34602-bib-0070] Starowicz, M. , and H. Zieliński . 2019. “Inhibition of Advanced Glycation End‐Product Formation by High Antioxidant‐Leveled Spices Commonly Used in European Cuisine.” Antioxidants (Basel) 8, no. 4: 100. 30991695 10.3390/antiox8040100 PMC6523868

[fsn34602-bib-0057] Ștefănescu, B. E. , K. Szabo , A. Mocan , and G. Crişan . 2019. “Phenolic Compounds From Five *Ericaceae* Species Leaves and Their Related Bioavailability and Health Benefits.” Molecules 24, no. 11: 2046. 10.3390/molecules24112046. 31146359 PMC6600139

[fsn34602-bib-0058] Stewart, L. K. , Z. Wang , D. Ribnicky , J. L. Soileau , W. T. Cefalu , and T. W. Gettys . 2009. “Failure of Dietary Quercetin to Alter the Temporal Progression of Insulin Resistance Among Tissues of C57BL/6J Mice During the Development of Diet‐Induced Obesity.” Diabetologia 52: 514–523. 10.1007/s00125-008-1252-0. 19142628 PMC2758024

[fsn34602-bib-0059] Tayyab, M. , M. Hanif , A. Rafey , et al. 2021. “UHPLC, ATR‐FTIR Profiling and Determination of 15 Lox, α‐Glucosidase, AGEs Inhibition and Antibacterial Properties of *Citrus* Peel Extracts; a Functional Food Based Approach.” Pharmaceutical Chemistry Journal 55: 138–148.

[fsn34602-bib-0060] Thornalley, P. J. , A. Langborg , and H. S. Minhas . 1999. “Formation of Glyoxal, Methylglyoxal and 3‐Deoxyglucosone in the Glycation of Proteins by Glucose.” Biochemsirty Journal 344, no. Pt 1: 109–116. PMC1220620 10548540

[fsn34602-bib-0061] Upadhyay, A. , E. Tuenter , A. Amin , et al. 2014. “5‐*O*‐Demethylnobiletin, a Polymethoxylated Flavonoid, From *Citrus depressa* Hayata Peel Prevents Protein Glycation.” Journal of Functional Foods 11: 243–249.

[fsn34602-bib-0062] Walle, T. 2007. “Methoxylated Flavones, a Superior Cancer Chemopreventive Flavonoid Subclass?” Semin Cancer Biology 17, no. 5: 354–362. 10.1016/j.semcancer.2007.05.002 PMC2024817 17574860

[fsn34602-bib-0103] Wang, F. , L. Chen , C. Hongping , C. Shiwei , and Y. Liu . 2019. “Analysis of Flavonoid Metabolites in Citrus Peels (*Citrus reticulata* “Dahongpao”) Using UPLC‐ESI‐MS/MS.” Molecules 24: 2680. 10.3390/molecules24152680. 31344795 PMC6696472

[fsn34602-bib-0064] Yee, N. , L. G. Benning , V. R. Phoenix , and F. G. Ferris . 2004. “Characterization of Metal‐Cyanobacteria Sorption Reactions: A Combined Macroscopic and Infrared Spectroscopic Investigation.” Environmental Science and Technology 38: 775–782. 14968864 10.1021/es0346680

[fsn34602-bib-0065] Zhang, J. Y. , Q. Zhang , H. X. Zhang , Q. Ma , J. Q. Lu , and Y. J. Qiao . 2012. “Characterization of Polymethoxylated Flavonoids (PMFs) in the Peels of ‘Shatangju’ Mandarin ( *Citrus reticulata* Blanco) by Online High‐Performance Liquid Chromatography Coupled to Photodiode Array Detection and Electrospray Tandem Mass Spectrometry.” Journal of Agriculture and Food Chemistry 60, no. 36: 9023–9034. 10.1021/jf302713c 22917253

